# Surveillance of close contacts of patients with infectious tuberculosis: a prospective cohort study

**DOI:** 10.1186/s13756-024-01419-z

**Published:** 2024-06-09

**Authors:** Zichun Ma, Shujuan Duan, Wei Wang, Rongmei Liu, Shanshan Li, Yuanyuan Shang, Xuxia Zhang, Jinfeng Yuan, Mengqiu Gao, Yu Pang

**Affiliations:** 1 Department of Bacteriology and Immunology, Beijing Chest Hospital, Beijing Tuberculosis and Thoracic Tumor Research Institute, Capital Medical University, Postal No 9, Beiguan Street, Tongzhou District, Beijing, 101149 People’s Republic of China; 2 Department of Tuberculosis, Beijing Chest Hospital, Capital Medical University/Beijing Tuberculosis and Thoracic Tumor Research Institute, Postal No 9, Beiguan Street, Tongzhou District, Beijing, 101149 People’s Republic of China

**Keywords:** Tuberculosis, Close contact, Risk factor, IGRA

## Abstract

**Background:**

A long-term follow-up of close contacts to monitor their infection status is essential to formulate a promising screening strategy. The study aimed to assess the dynamics of tuberculosis (TB) infection using Interferon-γ release assay (IGRA) and determine risk factors associated with TB infection.

**Methods:**

Definite TB patients were interviewed and their household contacts were screened for TB infection by IGRA during 12-month longitudinal investigation.

**Results:**

We included in our analyses 184 household contacts of 92 index TB patients. 87 individuals (47.3%) in contact group progressed to TB infection, of whom 86 developed into IGRA positive within 24 weeks. Close contacts with a higher age and comorbidities are easier to exhibit TB infection. Analysis showed that risk factors for becoming IGRA-positive individuals included residence, older age, comorbidities, BCG scar and high bacterial load. Contacts with BCG scar had a lower IGRA-positive rate.

**Conclusion:**

IGRA conversion generally occurs within 24 weeks after exposure. The TB transmission happens since subclinical TB stage and the presence of BCG scar is an independent protective factor reducing risk of TB infection among close contacts. Repeated IGRA tests are sensible to conducted among close contacts at 24 weeks after exposure to identify the IGRA-positive individuals.

**Supplementary Information:**

The online version contains supplementary material available at 10.1186/s13756-024-01419-z.

## Introduction

Tuberculosis (TB), caused by *Mycobacterium tuberculosis* (MTB) complex, remains a serious public health threat globally [1, 2]. According to the World Health Organization (WHO) estimates, in 2022 there were 10.6 million people with TB and 1.3 million deaths [3]. This disease has been strongly associated with poverty, overcrowding, malnutrition, and smoking [4]. Moreover, approximately one-third of the world’s population is believed to be latently infected with TB, termed as latent TB (LTBI). Although an estimated 10% of LTBI individuals develop active TB during their lifetime, the huge reservoir of potential future source of active TB poses a barrier towards international targets for achieving TB control [5, 6]. The management of LTBI, therefore, is of great importance for successful achievement of the END TB Strategy.

The transmission of TB occurs mainly via inhalation of airborne droplet nuclei carrying viable bacteria [2]. The risk of acquiring infection with MTB correlates with exposure duration to an infectious source [7]. The WHO recommends systematic monitoring of population groups at high-risk for TB exposure, including household contacts of TB-afflicted individuals and healthcare workers [8]. Specially, in comparison with the intensified infection control measures for healthcare workers, the household contacts are more prone to be infected with MTB, as well as at high risk of getting TB disease [9]. Thus, screening of persons in close contact with the index case is an important strategy to decrease the incidence, especially to identify infected individuals and take action to prevent the development the presence of active TB. In China, the close contacts of infectious people with TB are asked to be screened for TB infection after obtaining personal consent. Tuberculin skin test is the most frequently used method of inferring TB infection in clinical practice. Once identified, the preventive treatment is optional for individuals with TB infection, emphasizing the urgent need for developing efficient national strategies to improve the screening and management of TB infection among close contacts [10].

Routinely, an indirect immunological test is used to directly detect LTBI by ascertaining the reactivity of host lymphocytes to mycobacterial antigens, including *in viv*o response with tuberculin skin test (TST) or in vitro with INF-γ release assays (IGRAs) [5, 11, 12]. Specially, numerous previous studies have demonstrated that IGRAs outperform TST in identifying LTBI [13, 14]. The high costs of equipment and consumables for IGRAs make it difficult to access in many endemic settings [12, 15]. Even in high-income, low TB burden countries, the cross-sectional IGRA-based screening is usually conducted among close contacts. However, the development of adaptive immunity requires multiple weeks in individuals infected with MTB, and the delayed adaptive immune response would undoubtedly lead to “false-negative” IGRAs. A long-term follow-up of close contacts to monitor their infection status is essential to formulate a promising screening strategy. In the present study, we aimed to assess the dynamics of TB infection using IGRAs over a follow-up period of 1 year, and determine risk factors associated with TB infection in our setting.

## Materials and methods

### Study populations

Between July 2022 and March 2024, a prospective cohort study of TB household contacts was conducted, 92 local TB patients with confirmed pathogenic diagnosis result were recruited as index case. Their household contacts who more than 5 years old were enrolled.

### Contact investigation and contact tracing

**Fig. 1:**
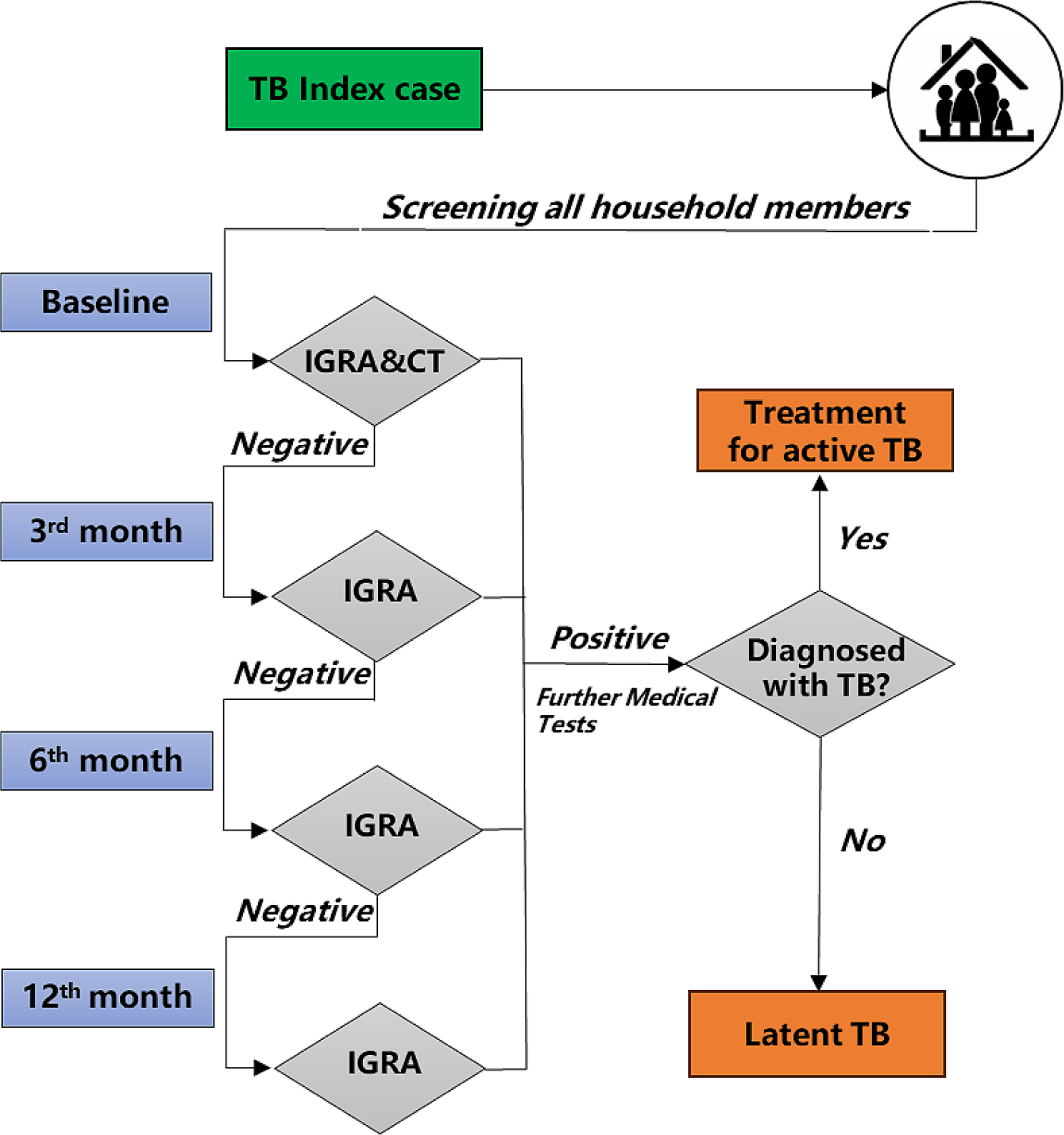
Workflow for screening the household contacts. CT, Computed Tomography; IGRA, Interferon-γ Release Assays; TB, Tuberculosis

“Index case” was defined as notified bacteria-positive (Xpert, smear and/or culture-positive) TB patient. At baseline, sociodemographic and clinical characteristics information was collected included of both index patients and the household contacts (HHCs), meanwhile, the HHCs were screened by IGRA and CT scan. All contacts have a CT in the first time-point. Contacts with symptoms of TB or positive IGRA tests or CT scan underwent a clinical evaluation in Beijing Chest hospital. If participants were identified as having TB disease, they would receive medical treatment. Those HHCs with negative IGRA results were then traced for the following 12 months. At the 3rd, 6th and 12th months, HHCs with negative IGRA results were evaluated by a clinical questionnaire and IGRA test. In this cohort, all new people with TB were confirmed by IGRA combined with radiography changes (Fig. 1) and were provided timely treatment.

### IFN-γ release assay (IGRA)

The commercial sandwich enzyme-linked immunosorbent assay (ELISA) kit (Leide, Guangzhou China) was used to assess interferon-gamma (IFN-γ) concentration according to the manufacturer (Leide, Guangzhou China). Three heparinized fresh blood samples in the volume of 0.6 mL each were added to three different tubes included in kit and incubated at 37 ◦C for 20 ± 2 h after gentle mixing. The first tube did not contain any stimulating agent and was used to determine the individual IFN-γ background; The second tube included MTB-specific antigen (ESAT-6 and CFP-10); the third one was the positive-control tube, which was used for controlling the stimulation ability. During the incubation, the stimulable immune cells were activated to release IFN-γ. After the incubation, the tubes were centrifuged at 4000× rpm for 5 min to obtain stimulated heparinized plasma, which could be used to determine the IFN-γ concentration. The values obtained in the three tubes were used to calculate the final concentration of IFN-γ (pg/mL) released by T lymphocytes due to their stimulation with specific antigen. The results were based on a borderline range recommended by the manufacturer, where a value ≥ 20.0 pg/mL was considered as positive result.

### Statistical analyses

The data were entered using a Microsoft Excel worksheet and analyzed by R studio (v4.1.2; R Core Team 2021). Descriptive statistics were used to describe study population characteristics. SPSS version 20.0 software (IBM Corp., Armonk, NY) was used for statistical analysis of the survival curve. Logistic regression analysis (univariate and multivariate analysis) was performed using lme4 package in R studio. Variables with univariate *P* value less than 0.02 in the univariate analysis were considered in the multivariable models. After using backward elimination, we kept variables if they had a 2-sided significance level less than 0.05. Statistically significant differences were defined using the chi-square or Fisher’s exact test.

### Ethics statement

Written informed consent was obtained from all index cases and HHCs. The study was approved by the Ethics Committee of the Beijing Chest Hospital, Capital Medical University (approval No.: YJS-2022-03). The information of all individuals involved in the study were anonymized.

## Result

### Demographic characteristics of close contacts and index patients

Characteristics of the 92 index patients and 184 close contacts enrolled in the study are shown in Tables 1 and 2. All close contacts completed a 12-month follow-up by the end of March 2024. At enrollment of the index patients, the section of median age was 48 years and 38.0% (*n* = 35) were older than 60 years of age. About 71.7% (*n* = 66) of the index patients were male. In addition, 38.0% (*n* = 35) patients had positive sputum culture and 28.3% (*n* = 26) had cavitation on chest radiography, only 14 (15.2%) index patients progressed to the calcification stage of TB (Table 1).

**Table 1:** Clinical and Demographic Characteristics of Index Tuberculosis

	Total (*n* = 92)
Sex	
Male	66 (71.7%)
Female	26 (28.3%)
Age	
5–20 years	3 (3.3%)
20–60 years	54 (58.7%)
> 60 years	35 (38.0%)
Xpert	
Positive	89 (96.7%)
Negative	3 (3.3%)
Sputum Culture	
Positive	35 (38.0%)
Negative	4 (4.3%)
Unknown	53 (57.6%)
Sputum Smear	
4+	2 (2.2%)
3+	23 (25.0%)
2+	13 (14.1%)
1+	29 (31.5%)
Unknown	25 (27.2%)
IGRAs^a^	
Positive	32 (34.8%)
Negative	5 (5.4%)
Unknown	55 (59.8%)
Tuberculous cavity	
Yes	26 (28.3%)
No	66 (71.7%)
Tuberculous calcification	
Yes	14 (15.2%)
No	78 (84.8%)

^a^ IGRAs, Interferon-γ release assays

**Table 2:** Univariate Analysis of Risk Factors for MTB infection of TB Contacts

Risk factor	Negative IGRAs	Positive IGRAs	Total	*P* value
	(*n* = 97)	(*n* = 87)	(*n* = 184)	
Sex				0.685
Male	34 (35.1%)	33 (37.9%)	67 (36.4%)	
Female	63 (64.9%)	54 (62.1%)	117 (63.6%)	
Age				*0.013
5–20 years	5 (5.2%)	10 (11.5%)	15 (8.2%)	
20–60 years	80 (82.5%)	55 (63.2%)	135 (73.4%)	
> 60 years	12 (12.4%)	22 (25.3%)	34 (18.5%)	
Residence ^a^				*0.016
Local	68 (70.1%)	46 (52.9%)	114 (62.0%)	
Migrant	29 (29.9%)	41 (47.1%)	70 (38.0%)	
Ethnic				0.845
Han nationality	91 (93.8%)	81 (93.1%)	172 (93.5%)	
other nationality	6 (6.2%)	6 (6.9%)	12 (6.5%)	
Education				0.641
Junior high school and below	26 (26.8%)	28 (32.2%)	54 (29.3%)	
high school	20 (20.6%)	19 (21.8%)	39 (21.2%)	
College degree and above	51 (52.6%)	40 (46.0%)	91 (49.5%)	
BMI (kg/m^2^) ^b^				0.604
< 18.5	6 (6.2%)	8 (9.2%)	14 (7.6%)	
18.5–24	39 (40.2%)	30 (34.5%)	69 (37.5%)	
> 24	52 (53.6%)	49 (56.3%)	101 (54.9%)	
HIV				0.697
Negative	20 (20.6%)	20 (23.0%)	40 (21.7%)	
Unknown	77 (79.4%)	67 (77.0%)	144 (78.3%)	
Comorbidity				**0.006
No	84 (86.6%)	61 (70.1%)	145 (78.8%)	
Yes	13 (13.4%)	26 (29.9%)	39 (21.2%)	
Frequency of colds				0.291
0–1	72 (74.2%)	70 (80.5%)	142 (77.2%)	
2–3	25 (25.8%)	16 (18.4%)	41 (22.3%)	
≥ 4	0 (0.0%)	1 (1.1%)	1 (0.5%)	
BCG scar				**0.005
No	16 (16.5%)	30 (34.5%)	46 (25.0%)	
Yes	81 (83.5%)	57 (65.5%)	138 (75.0%)	
History of tuberculosis				0.065
No	97 (100.0%)	84 (96.6%)	181 (98.4%)	
Yes	0 (0.0%)	3 (3.4%)	3 (1.6%)	
History of tuberculosis in family				0.192
No	2 (2.1%)	5 (5.7%)	7 (3.8%)	
Yes	95 (97.9%)	82 (94.3%)	177 (96.2%)	
Relationship with the index case				0.252
Conjugal	29 (29.9%)	29 (33.3%)	58 (31.5%)	
Children	43 (44.3%)	38 (43.7%)	81 (44.0%)	
Grandchildren	12 (12.4%)	4 (4.6%)	16 (8.7%)	
Other	13 (13.4%)	16 (18.4%)	29 (15.8%)	
Degree of closeness				0.358
≤ 1 h per day	18 (18.6%)	12 (13.8%)	30 (16.3%)	
1–3 h per day	13 (13.4%)	19 (21.8%)	32 (17.4%)	
3–5 h per day	17 (17.5%)	13 (14.9%)	30 (16.3%)	
5–8 h per day	6 (6.2%)	2 (2.3%)	8 (4.3%)	
≥ 8 h per day	43 (44.3%)	41 (47.1%)	84 (45.7%)	
Medication				0.912
No	95 (97.9%)	85 (97.7%)	180 (97.8%)	
Yes	2 (2.1%)	2 (2.3%)	4 (2.2%)	
Degree of Xpert (Index cases)				**0.000
Low^c^	44 (45.4%)	35 (40.2%)	79 (42.9%)	
Medium	48 (49.5%)	39 (44.8%)	87 (47.3%)	
High	5 (5.1%)	13 (14.9%)	18 (9.8%)	
Index Situation				
Subclinical TB^d^	9 (9.3%)	8 (9.2%)	17 (9.2%)	1.000
Active TB	88 (90.7%)	79 (90.8%)	167 (90.8%)	

^a^ Residence of the follow-up contacts divided into two categories: One was “local”, that is, the follow-up personnel whose domicile was Beijing; The other is “Migrant” whose domicile is not in Beijing

^b^ BMI, body mass index

^c^ Low degree of Xpert included extremely low and low TB DNA detected

^d^ Subclinical TB was defined as bacteriologically-confirmed but negative on symptom screening

Among the contacts, approximately 63.6% (*n* = 117) of the close contacts were female, and 54.9% (*n* = 101) were categorized as overweight or obese. Nearly none of the follow-up contacts had a history of tuberculosis, while three-quarters of the close contacts (*n* = 138) exhibited a scar from *Mycobacterium bovis* BCG vaccination. 20% of the contact study population (*n* = 39) had additional comorbidities, such as diabetes mellitus, cardiopathy, hypertension, and other diseases (Table 2).

### Progression of household contacts

**Fig. 2:**
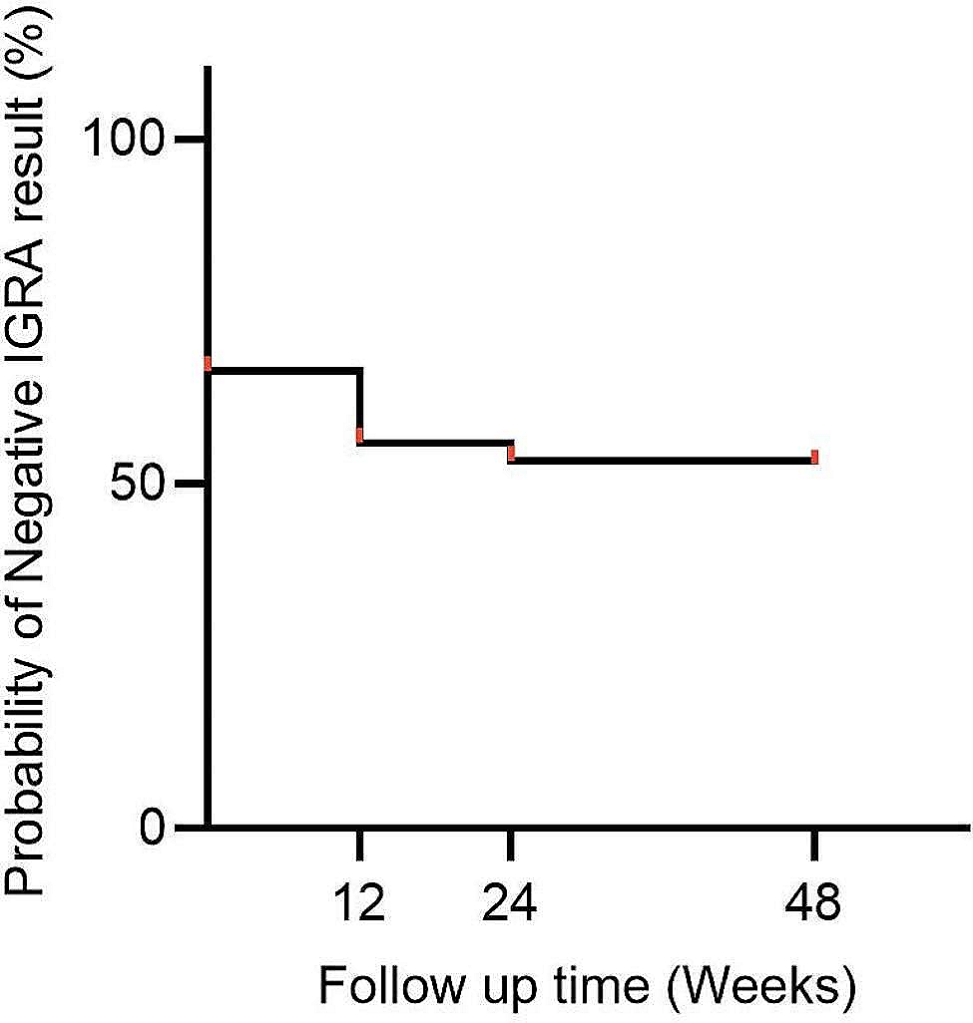
Cumulative IGRA negative rate in TB household contacts at baseline and follow-up visits. IGRA, Interferon-γ Release Assays

Over the course of more than one year of follow-up, 62 individuals (33.7%) within the contact group had positive IGRA results at baseline and 25 individuals (13.6%) within the contact group converted to a positive IGRA result. Thus 81 (44.0%) of the contacts had a positive IGRA result within 12 weeks (Fig. 2). IGRA conversion predominantly took place within 24 weeks (86/87, 98.9%) from the commencement of this follow-up study. Only one close contact converted to IGRA-positive result after 24 weeks (48 weeks).

### Risk and protective effects associated with positive Interferon-γ release assay

In univariate analyses of positive IGRA result among close contacts, notable distinctions emerged between negative IGRA contacts and positive IGRA contacts across several factors. These factors encompassed residence (*P* = 0.016), advanced age (*P* = 0.013), comorbidities (*P* = 0.006), presence of BCG scar (*P* = 0.005), and extent of Xpert (*P*<0.001). In univariate analyses of IGRA conversion among close contacts, there was no significant difference between HHCs who were negative at baseline and subsequently had IGRA conversion and HHCs where IGRA remained negative by 48 weeks (Table S1).

In a multivariable model that encompassed HHCs with positive and negative IGRA results, several risk factors and protective factors for positive results emerged. These included migrant (defined as domicile was not in Beijing) (OR [95%CI]: 2.621[1.363–5.149], *P* = 0.004), advanced age (OR [95%CI]: 3.449[1.063–12.479], *P* = 0.045), comorbidities (OR [95%CI]: 2.726[1.199–6.412], *P* = 0.018), presence of BCG scar (OR [95%CI]: 0.424[0.199–0.877], *P* = 0.022) (Table 3).

**Table 3:** Multivariable analysis of risk factors for MTB infection of TB contacts

Risk factor	*n* = 184			
	OR^a^	95%CI^b^	*P* value	
Age				
< 20 years	Referent			
20–60 years	3.449	1.063–12.479	*0.045	
> 60 years	2.287	0.968–5.549	0.061	
Residence ^c^	2.621	1.363–5.149	**0.004	
Comorbidity ^d^	2.726	1.199–6.412	*0.018	
BCG Scar	0.424	0.199–0.877	*0.022	
Degree of Xpert (Index cases)				
Low^e^	Referent			
Medium	3.120	0.943–10.320	0.062	
High	0.302	0.090–1.009	0.052	

^a^ OR: Odds ratio

^b^ CI: Confidence Interval

^C^ Residence of the follow-up contacts divided into two categories: One was “local”, that is, the follow-up personnel whose domicile was Beijing; The other is “Migrant” whose domicile is not in Beijing. The reference group is the follow-up personnel whose domicile was Beijing

^d^ Comorbidities included diabetes mellitus, cardiopathy, hypertension, and a few other diseases

^e^ Low degree of Xpert included extremely low and low TB DNA detected

Moreover, individuals with BCG scars among the contacts exhibited a reduced positive IGRA rate (57/138, 41.3%) in contrast to those without BCG scars (30/46, 65.2%). This disparity suggests that contacts possessing a BCG scar experienced a diminished risk of tuberculosis (Table 3). The rate of positive IGRA in close contacts of patients with subclinical symptoms (defined as bacteriologically confirmed but negative on symptom screening) closely mirrored the rate observed in the close contacts of patients with active tuberculosis (47.1% for the symptomatic patient group versus 47.3% for the asymptomatic patient group, *P* = 1.000) (Table 2).

## Discussion

Early identification and intervention for TB infections among close contacts decrease the likelihood of TB transmission within the community [16]. Conventional screening of contacts of people with infectious TB at baseline inevitably results in underestimation of TB infections due to time lags in developing adaptive immune responses against MTB. In this study, our data demonstrated that IGRA conversion majorly occurred within 24 weeks since the investigation began, and only one close contact showed a positive IGRA response at 48 weeks. In a previous cohort study in Korea, Lee and colleagues found that IGRA conversion generally occurred within 22 weeks after exposure, and no conversion was noted 30 weeks after the outbreak investigation [17]. The different intervals of IGRA conversion could be explained by individual diversity in the generation of a lymphocyte memory immune response. Although the memory T cell formation generally takes weeks, this duration varies across individuals, which depends on the immune status of the host organism, as well as pathogenicity of tubercle bacilli. The inclusion of close contact in one TB outbreak may not reflect the diversity of MTB isolates, thereby resulting in biased estimation of IGRA conversion. Previous guidelines endorse repeating the IGRA test at 6–10 weeks after TB exposure among close contacts with a negative IGRA result at initial [18, 19]. Based on the results of our study, repeated IGRA tests should be conducted among close contacts at 24 weeks after exposure to identify the maximum individuals infected with MTB.

Subclinical TB poses a substantial challenge to TB control. Individuals with subclinical TB would be missed if the screening approach had included patients with clinical symptoms suggestive of active TB. In this study, 17 subclinical index cases were identified. Several modeling studies have demonstrated that the evaluation and diagnosis of subclinical index cases of TB disease could contribute to retarding MTB transmission [20]; however, this hypothesis has not emerged from observed data, but from modeling analysis. The results of the present study revealed that the TB transmission occurred from exposure to individuals with subclinical TB. Theoretically, the cycle of TB transmission requires inhalation of an infectious droplet nucleus into the alveolus of a TB-naïve person. For most active TB patients, coughing is assumed to be the main way to produce infectious airborne droplet nuclei. Interestingly, a recent report of face-mask sampling indicated that cough was not necessary for MTB transmission, and the exhaled MTB output was not associated with cough frequency and disease severity [21]. Similarly, Wang and coresearchers identified potential TB transmission among detainee at subclinical TB stage [22]. We consider that subclinical index patients still have atypical clinical symptoms. Breathing, talking and other respiratory maneuvers may play an important role in TB transmission on a population scale. Our findings, combined with previous evidence, highlights an urgent need to address subclinical presentations of the disease.

Although BCG vaccination protects against miliary and meningeal TB in infants and children, its protective effect against adult TB is controversial. In multiple randomized controlled trials and observational studies, the protective effect of BCG vaccination ranges from negative to 100% [23]. In the present study, our data demonstrated that the presence of BCG scar, a useful indicator of an individual’s immune response to BCG vaccination, was an independent protective factor reducing risk of TB infection among close contacts [23], suggesting that BCG vaccination may provide consistent protection in adults. In addition, we found significantly increased risk of TB infection among elderly close contacts. This phenomenon may reflect poor innate immune responses against MTB in these individuals and a higher prevalence of remote infection (infection from the past). Previous experimental studies support our findings at the cellular level, the alveolar macrophages from elderly mice were more permissive to MTB growth and survival [24, 25]. The failure of early clearance of invasive MTB would undoubtedly facilitate antigen presentation and development of adaptive immunity against MTB. In order to achieve the EndTB Strategy target to eliminate the TB epidemic by 2035, more effective vaccines are also urgently needed to protect these vulnerable populations from new MTB infections.

Our study had several obvious limitations. First, our data should be interpreted with caution, as the results came from HIV-naïve populations; bias in the analysis may occur when our conclusion is extended to HIV-positive populations. Second, because the majority of MTB-infected individuals would not ultimately progress to TB disease, we did not perform genotyping to distinguish transmission sources. Additionally, the TST could boost the response of subsequent IGRA test [26, 27], thereby resulting in false-positive IGRA results. Taken together, the infection rate among close contacts may be overestimated in our analysis. Third, adaptive immunity always takes approximately 3–8 weeks after MTB infection [28]. In our cohort, 95.8% (23/24) of close contact showed IGRA conversion by 24 weeks after exposure. Besides the delayed immunological response to TB infection, one plausible explanation for this observation may be associated with the subsequent exposure to a unrecorded index case. Fourth, the time lag between onset of TB symptoms and enrolment on treatment is an important determinant of spread of TB infection within household contacts. However, considering that the onset self-reported symptoms of the index patient is a subjective measure and many factors influence a patients answer, this time log was not recorded in our analysis, which hampered our ability to provide an accurate estimate of window period of conversion after exposure to MTB. Fifth, although the benefits of TB preventive therapy have been addressed by several TB contact cohort studies [29–31], the majority of individuals refuse preventive therapy in China, which limits our ability to evaluate the efficacy of this intervention for preventing development of active disease. Finally, our data came from a single pilot, potentially limiting the generalizability of our results to other settings.

In conclusion, our data demonstrates that IGRA conversion generally occurs within 24 weeks after exposure. In addition, the results of the present study reveal that the TB transmission can happen during a subclinical TB stage. The presence of BCG scar is an independent protective factor reducing risk of TB infection among close contacts; whereas we identified significantly increased risk of TB infection among the elderly individuals. Based on the results of the represent study, repeated IGRA tests should be conducted among close contacts at 24 weeks after exposure to identify the maximum individuals infected with TB.

### Electronic supplementary material

Below is the link to the electronic supplementary material.

Supplementary Material 1

## Data Availability

No datasets were generated or analysed during the current study.
