# Supplementary material for: Surveillance of close contacts of patients with infectious tuberculosis: a prospective cohort study
Source: Antimicrob Resist Infect Control. 2024 Jun 9;13:59. doi: 10.1186/s13756-024-01419-z (PMC11163748; doi:10.1186/s13756-024-01419-z)
Supplement: Supplementary file 1 — Supplementary Material 1 [file 13756_2024_1419_MOESM1_ESM.docx]

Table S1 Univariate Analysis of Risk Factors for IGRA conversion

| Risk factor | IGRA  Converter | IGRA  Persistently Negative | | Total | | *P* value |
| --- | --- | --- | --- | --- | --- | --- |
|  | （n=22） | （n=87） | （n=109） | | |  |
| Sex |  |  |  | | 0.798 | |
| Male | 9 (40.9%) | 33 (37.9%) | 42 (38.5%) | |  | |
| Female | 13 (59.1%) | 54 (62.1%) | 67 (61.5%) | |  | |
| Age |  |  |  | | 0.248 | |
| < 20 years | 1 (4.5%) | 10 (11.5%) | 11 (10.1%) | |  | |
| 20–60 years | 18 (81.8%) | 55 (63.2%) | 73 (67.0%) | |  | |
| > 60 years | 3 (13.6%) | 22 (25.3%) | 25 (22.9%) | |  | |
| Residence^a^ |  |  |  | | 0.888 | |
| Local | 12(54.5%) | 46 (52.9%) | 58 (53.2%) | |  | |
| Migrant | 10 (45.5%) | 41 (47.1%) | 51 (46.8%) | |  | |
| Ethnic |  |  |  | | 0.688 | |
| Han nationality | 21 (95.5%) | 81 (93.1%) | 102 (93.6%) | |  | |
| other nationality | 1 (4.5%) | 6 (6.9%) | 7 (6.4%) | |  | |
| Education |  |  |  | | 0.668 | |
| Junior high school and below | 5 (22.7%) | 28 (32.2%) | 33 (30.3%) | |  | |
| high school | 6 (27.3%) | 19 (21.8%) | 25 (22.9%) | |  | |
| College degree and above | 11 (50.0%) | 40 (46.0%) | 51 (46.8%) | |  | |
| BMI (kg/m^2^) ^b^ |  |  |  | | 0.561 | |
| < 18.5 | 1 (4.5%) | 8 (9.2%) | 9 (8.3%) | |  | |
| 18.5-24 | 6 (27.3%) | 30 (34.5%) | 36 (33.0%) | |  | |
| > 24 | 15 (68.2%) | 49 (56.3%) | 64 (58.7%) | |  | |
| HIV |  |  |  | | 0.391 | |
| Negative | 7 (31.8%) | 20 (23.0%) | 27 (24.8%) | |  | |
| Unknown | 15 (68.2%) | 67 (77.0%) | 82 (75.2%) | |  | |
| Comorbidity |  |  |  | | 0.506 | |
| No | 17 (77.3%) | 61 (70.1%) | 78 (71.6%) | |  | |
| Yes | 5 (22.7%) | 26 (29.9%) | 31 (28.4%) | |  | |
| Frequency of colds |  |  |  | | 0.879 | |
| 0-1 | 18 (81.8%) | 70 (80.5%) | 88 (80.7%) | |  | |
| 2-3 | 4 (18.2%) | 16 (18.4%) | 20 (18.3%) | |  | |
| ≥4 | 0 (0%) | 1 (1.1%) | 1 (1.0%) | |  | |
| BCG scar |  |  |  | | 0.574 | |
| No | 9 (40.9%) | 30 (34.5%) | 39 (35.8%) | |  | |
| Yes | 13 (59.1%) | 57 (65.5%) | 70 (64.2%) | |  | |
| History of tuberculosis |  |  |  | | 0.576 | |
| No | 20 (90.9%) | 84 (96.6%) | 104 (95.4%) | |  | |
| Yes | 2 (9.1%) | 3 (3.4%) | 5 (4.6%) | |  | |
| History of tuberculosis in family |  |  |  | | 0.205 | |
| No | 3 (13.6%) | 5 (5.7%) | 8 (7.3%) | |  | |
| Yes | 19 (86.4%) | 82 (94.3%) | 101 (92.7%) | |  | |
| Relationship with the index case |  |  |  | | 0.696 | |
| Conjugal | 8 (36.4%) | 29 (33.3%) | 37 (33.9%) | |  | |
| Children | 11 (50.0%) | 38 (43.7%) | 49 (45.0%) | |  | |
| Grandchildren | 0 (0%) | 4 (4.6%) | 4 (3.7%) | |  | |
| Other | 3 (13.6%) | 16 (18.4%) | 19 (17.4%) | |  | |
| Degree of closeness |  |  |  | | 0.815 | |
| ≤ 1 hour per day | 3 (13.6%) | 12 (13.8%) | 15 (13.8%) | |  | |
| 1-3 hours per day | 3 (13.6%) | 19 (21.8%) | 22 (20.2%) | |  | |
| 3-5 hours per day | 5 (22.7%) | 13 (14.9%) | 18 (16.5%) | |  | |
| 5-8 hours per day | 1 (4.5%) | 2 (2.3%) | 3 (2.8%) | |  | |
| ≥ 8 hours per day | 10 (45.5%) | 41 (47.1%) | 51 (46.7%) | |  | |
| Medication |  |  |  | | 0.130 | |
| No | 20 (90.9%) | 85 (97.7%) | 105 (96.3%) | |  | |
| Yes | 2 (9.1%) | 2 (2.3%) | 4 (3.7%) | |  | |
| Degree of Xpert (Index cases) |  |  |  | | 0.639 | |
| Low^c^ | 11 (50.0%) | 35 (40.2%) | 46 (42.2%) | |  | |
| Medium | 9 (40.9%) | 39 (44.8%) | 48 (44.0%) | |  | |
| High | 2 (9.1%) | 13 (14.9%) | 15 (13.8%) | |  | |
| Index Situation |  |  |  | | 0.537 | |
| Subclinical TB^d^ | 3 (13.6%) | 8 (9.2%) | 11 (10.1%) | |  | |
| Active TB | 19 (86.4%) | 79 (90.8%) | 98 (89.9%) | |  | |

^a^ Residence of the follow-up contacts divided into two categories: One was “local”, that is, the follow-up personnel whose domicile was Beijing; The other is “Migrant” whose domicile is not in Beijing.

^b^ BMI, body mass index.

^c^ Low degree of Xpert included extremely low and low TB DNA detected.

^d^ Subclinical TB was defined as bacteriologically-confirmed but negative on symptom screening.
